# Long non-coding RNAs and their potential functions in Ligon-lintless-1 mutant cotton during fiber development

**DOI:** 10.1186/s12864-019-5978-5

**Published:** 2019-08-19

**Authors:** Haron Salih, Wenfang Gong, Shoupu He, Wang Xia, Magwanga Richard Odongo, Xiongming Du

**Affiliations:** 1 Institute of Cotton Research, Chinese Academy of Agricultural Science (ICR, CAAS)/State Key Laboratory of Cotton Biology, Anyang, 455000 China; 2 Zalingei University, Central Darfur, Sudan

**Keywords:** Identification, Comparative analysis, LncRNAs, Ligon-lintless-1 mutant, Wild-type, Cell fiber development

## Abstract

**Background:**

Long non-coding RNAs (LncRNAs) are part of genes, which are not translated into proteins and play a vital role in plant growth and development. Nevertheless, the presence of LncRNAs and how they functions in Ligon-lintless-1 mutant during the early cessation of cotton fiber development are still not well understood. In order to investigate the function of LncRNAs in cotton fiber development, it is necessary and important to identify LncRNAs and their potential roles in fiber cell development.

**Results:**

In this work, we identified 18,333 LncRNAs, with the proportion of long intergenic noncoding RNAs (LincRNAs) (91.5%) and anti-sense LncRNAs (8.5%), all transcribed from Ligon-lintless-1 (Li1) and wild-type (WT). Expression differences were detected between Ligon-lintless-1 and wild-type at 0 and 8 DPA (day post anthesis). Pathway analysis and Gene Ontology based on differentially expressed LncRNAs on target genes, indicated fatty acid biosynthesis and fatty acid elongation being integral to lack of fiber in mutant cotton. The result of RNA-seq and RT-qPCR clearly singles out two potential LncRNAs, LNC_001237 and LNC_017085, to be highly down-regulated in the mutant cotton. The two LncRNAs were found to be destabilized or repressed by ghr-miR2950. Both RNA-seq analysis and RT-qPCR results in Ligon-lintless-1 mutant and wild-type may provide strong evidence of LNC_001237, LNC_017085 and ghr-miR2950 being integral molecular elements participating in various pathways of cotton fiber development.

**Conclusion:**

The results of this study provide fundamental evidence for the better understanding of LncRNAs regulatory role in the molecular pathways governing cotton fiber development. Further research on designing and transforming LncRNAs will help not only in the understanding of their functions but will also in the improvement of fiber quality.

**Electronic supplementary material:**

The online version of this article (10.1186/s12864-019-5978-5) contains supplementary material, which is available to authorized users.

## Background

Cotton fiber is one of the most important renewable resources of the textile industry worldwide. Cotton fiber develops from a single cell, and the nature of cell elongation provides an excellent model for testing gene expressions that relate to fiber development [1]. Fiber initiation occurs at the anthesis period, it emerges from the ovule epidermal cells [2, 3]. During the initiation period of cotton fiber development, only about 25% of cells on the ovule epidermis develop into a spinnable cotton fiber [1]. The cotton fiber developmental process is divided into 4 overlapping developmental stages: fiber initiation, fiber cell elongation, secondary cell wall formation and fiber cell maturation [1]. Initiation, elongation and secondary cell wall of cotton fiber have a great impact on the quantity, length and fineness of fiber, which are the main factors determining the lint yield quality [3]. The elongation stage of cotton fiber begins immediately after initiation stage and continues for around 3 weeks before the fiber cell switches to intensive deposition of secondary cell wall [4, 5]. Cotton fiber elongation depends on the genotype and prevailing environmental conditions [5]. Fiber developmental stage is controlled by a multi-complex interaction of genes rather than one gene effect, but the lack of evidence at molecular level regarding the network of transcriptional regulatory elements and genes that relate to cotton fiber development is one of the key setbacks in understanding the molecular components to improve fiber length [6]. Cotton fiber mutants are valuable materials for studying the molecular genetics and physiological processes of cell fiber development [7–11]. Recently, several fiber mutants have been revealed and used as model plants to study cotton fiber development, one of which is the monogenic and dominant mutant also known as the Ligon-lintless-1 [12]. The Ligon-lintless-1 mutant has an abnormal morphological appearance including, distorted leaves, stem and extreme reduction in the length of lint fiber about 4–6 mm at maturity stage [12]. Previous studies showed that the Li1 gene is located in the Dt sub-genome of *Gossypium hirsutum* on chromosome 22 (Dt 04) [13–15]. Gene expression analysis in the ovule of Ligon-lintless-1 mutant as compared to the wild-type, only few genes showed differential expression during the initiation stage of cotton fiber development [16, 17]. High expression levels of some secondary cell wall synthesis-related genes, such as tubulin, sucrose synthase and expansin were significantly expressed in Ligon-lintless-1 at the early stages of fiber development. However, they were highly expressed in wild-type cotton during the fiber elongation stage, probably elucidating the mechanism underlying the genotype of the Ligon-lintless-1 mutant [7, 15]. Previous studies using microarray technique and RNA-seq methods have attempted to identify key genes, which involved in regulating fiber elongation in the Ligon-lintlees-1 mutant [7, 17–20]*.* To understand the phenomenon of early cessation of fiber elongation in Ligon-lintless-1 mutant as compared to the wild-type, it is paramount to look into the molecular mechanisms or elements that are required to regulate cotton fiber development.

Long non-coding RNAs (LncRNAs) are defined as part of a non-coding RNAs longer than 200 bp (base pair) that do not have the capacity for coding proteins, which are involved in regulation of many biological regulatory processes [21]. According to their genomic location and context, LncRNAs can be divided into long noncoding natural antisense transcripts (LncNATs), long intergenic non-coding RNAs (LincRNAs), overlapping LncRNAs and long intronic non-coding RNAs [22]. Recent studies on plant LncRNAs have linked them to the vital biological processes including; controlling flowering period [23], gene silencing mechanism, abiotic stress tolerance, important developmental pathways [24–27] and cotton fiber development [28]. Many functions of plant LncRNAs are largely unknown except some LncRNAs such as COOLAIR (cold induced long antisense intragenic RNA), COLDAIR (cold assisted intronic noncoding RNA) and PHOSPHATE1. COOLAIR and COLDAIR play the important role in regulating vernalization in Arabidopsis [29] while PHOSPHATE1 regulates phosphate homeostasis and control photoperiod genic male sterility in rice [30]. In previous studies, several LncRNAs have been found to be involved in the regulation of epigenetic like RNA-directed DNA methylation and chromatin modification [28]. Recent investigation on noncoding RNAs in Ligon-lintless-1 and its wild-type is only limited to short noncoding RNAs (microRNAs) [31]. Naoumkina et al. 2016, identified an important small RNAs (microRNAs) expressed during fiber development in both Ligon-lintles-1 and Ligon-lintless-2 mutants as compared to their wild-type. The molecular mechanisms or elements underlying the LncRNAs related to cotton fiber development during initiation and early elongation stages are not well-known, thus using Ligon-lintless-1 mutant and its wild-type to identify and analyze differentially expressed long noncoding RNAs, which will provide a new dimension of understanding the cotton fiber development process. In this study, the sequencing of long non-coding RNA libraries constructed from cotton fiber development stages of Ligon-lintless-1 and wild-type was done. The identification of LncRNAs was analyzed in reference to *Gossypium hirsutum* TM-1 genome. We identified a total of 18,333 LncRNAs through five steps of filtration, of which 91.5% were LincRNAs and 8.5% were anti-sense LncRNAs. The functional prediction of long noncoding RNAs (LncRNAs) and their expressions as involved in cotton fiber development were examined. We investigated putative functional LncRNA candidates by differential expression analysis and co-expression network construction during cotton fiber development between Ligon-lintless-1 mutant and its wild-type.

## Results

### Identification of long noncoding RNAs in Ligon-lintless-1 and wild-type

**Fig. 1:**
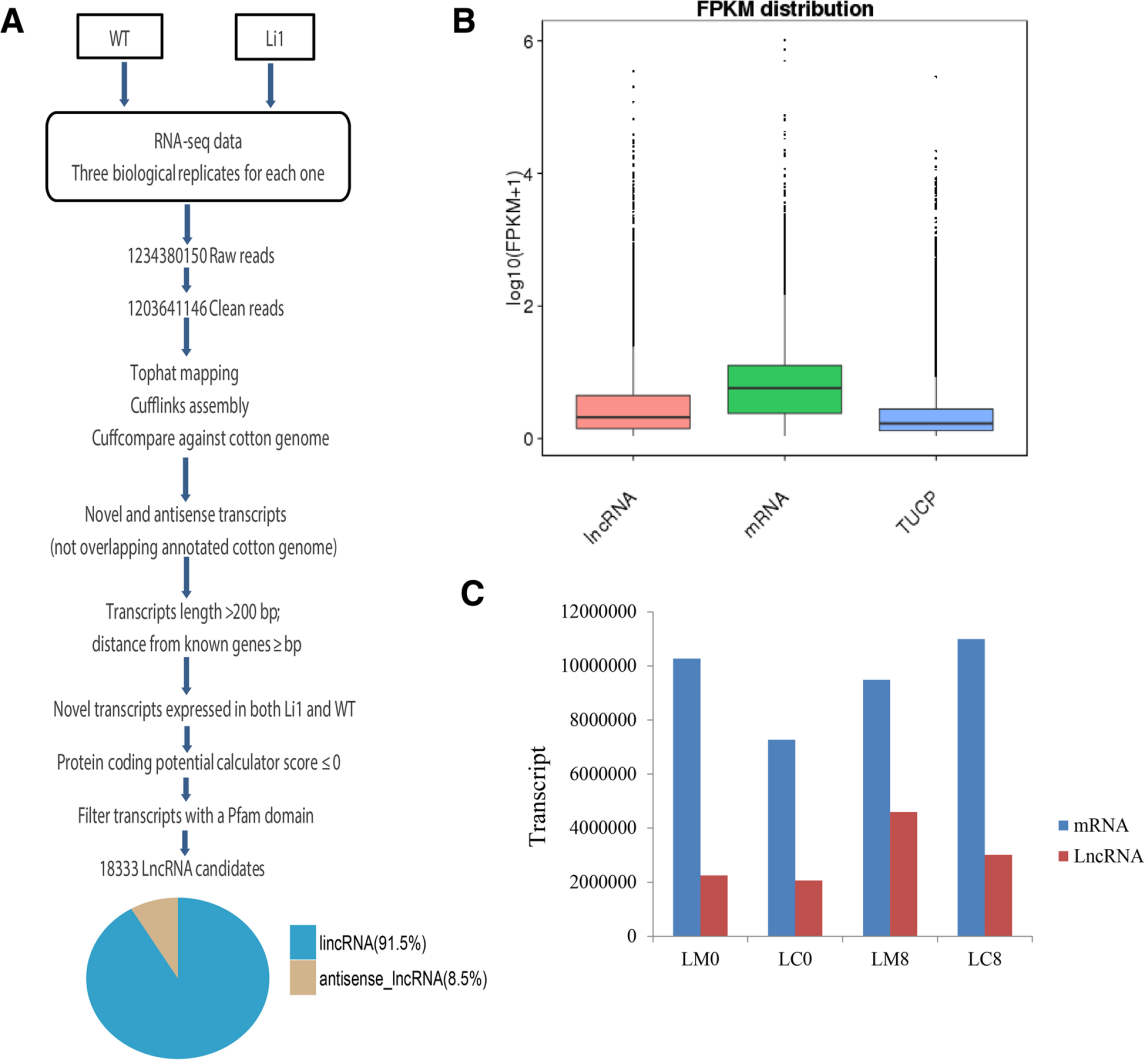
Scheme used for identification of LncRNAs in upland cotton. **a** The Pipeline was used to identify LncRNAs in upland cotton. **b** The distribution of LncRNAs, mRNAs and TUCP in upland cotton. **c** The number of expressed LncRNA and protein-coding transcripts in each cotton fiber stage

Cotton fiber initiation and rapid elongation stages are crucial stages with greater impact on fiber quantity and fiber length. It is essential to understand the various molecular pathways involved in the development of cotton fiber. Several proteins or genes have been identified with functional roles in fiber development [16, 20, 28, 32, 33]. For the identification of LncRNAs in Ligon-lintless-1 and wild-type, two critical stages of fiber development, initiation and elongation stages which are represented as 0 and 8 DPA were the points of investigation. Biological triplicates were done through the whole Transcriptome by Illumina sequencing, generating about 1.2 billion clean reads (Fig. 1a and Additional file 1: Table S1). Each clean read (RNA-seq clean data) was mapped to the entire *G. hirsutum* genome (TM-1) [34] independently using both Tophat2 (v2.0.9) software [35] and Bowtie 2 [36]. The transcripts generated from each RNA-seq data were assembled using both Cufflinks (v 2.1.1) [37] and Scripture (beta2) software [38]. In order to reduce transcriptional isoforms noise, only those transcripts assembled and were found in two or more samples by two tools reserved for subsequent analyses. All transcripts were pooled and merged to make the final transcriptome using Cuffmerge [37]. After generation of the final transcriptome, Cufflinks was employed to reconstruct the cotton transcriptome followed by transcript abundance assembly and analysis of differential isoform. Five stepped filtering out of the frequently expressed transcripts with FPKM scores < 0.5 (2 for single-exon transcripts) in all samples, transcripts without strand sense information and all transcripts that overlapped with annotated genes were removed; we recovered 78.39% of mRNAs in the dataset. The efficiency recovery of known protein-coding genes revealed that the preformed dataset was suitable for the retrieval of novel transcribed regions of the upland cotton genome (Additional file 1: Table S1). We only retained novel transcripts with the following specifications; not overlapping with known genes in a sense, longer than 200 nucleotides and expressed (for multiple-exon transcripts FPKM ≥0.5 and for single-exon transcripts FPKM ≥2). We further estimated the coding potential for the remaining transcripts using Coding Potential Calculator (CPC) [39] and Coding-Non-Coding Index (CNCI) [40] to predict the transcript coding potential. Only, the transcripts with CPC scores > 0 were removed (Fig. 1a). Furthermore, we used HMMER to confirm removal of protein-coding transcripts and checking of each transcript with CPC and CNCI score < 0 in all three reading frames to eliminate transcripts which encoded any protein domains family belonging to Pfam database [41]. Finally, we identified 18,333 constantly expressed LncRNAs, with proportions of 16,777 of LincRNAs and 1,556 of antisense LncRNAs, counting for 91.5 and 8.5% respectively (Fig. 1a and Additional file 1: Table S1).

### The distribution of LncRNAs and mRNAs in the upland cotton genome

**Fig. 2:**
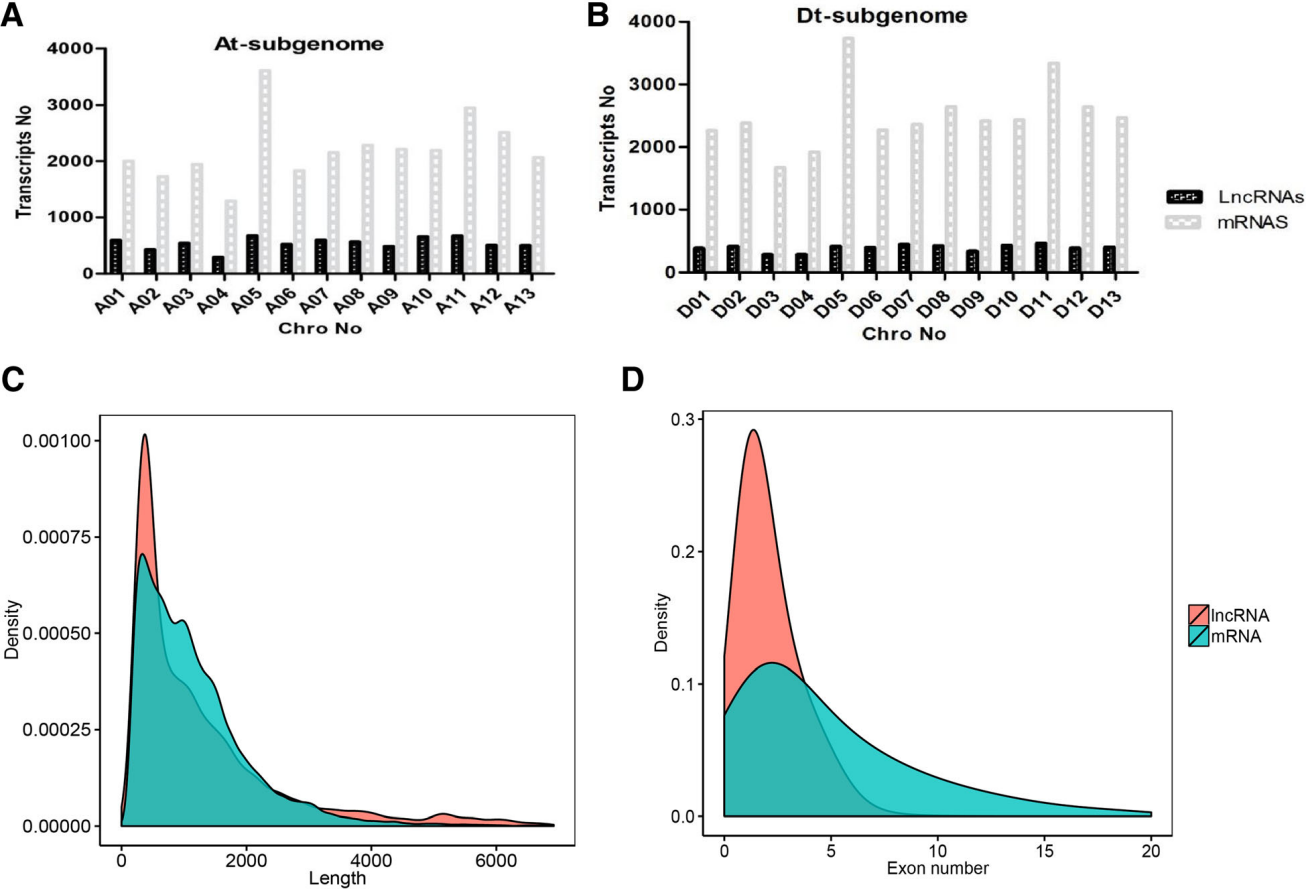
Characterization of LncRNAs transcribed from upland cotton. **a** and **b** The distribution of LncRNA and protein-coding transcripts in the At-subgenome and Dt-subgenome, respectively (**c**) Length distribution of LncRNAs and protein-coding transcripts. **d** Exon number distribution per the transcript of LncRNAs and protein-coding transcripts

To further investigate the distribution of LncRNAs and mRNAs in upland cotton, the sequence of the upland cotton genome provided a valuable tool [34]. We observed the distribution of LncRNAs on the upland cotton genome and identified 7,064, 5,089 and 6,280 LncRNAs, which were transcribed from the At-sub genome, Dt-sub genome and scaffolds (undefined chromosomes), respectively (Fig. 2a & b). In addition, we found that the lengths of LncRNAs were largely varied from 200 to 3,589 bp and transcribed from all upland cotton chromosomes and scaffolds. However, a total of 28,749, 35,514 and 9,219 mRNAs were transcribed from the At-genome, Dt-genome and scaffolds, respectively (Fig. 2a & b). Generally, mRNAs lengths varied from 150 to 428,268 bp and also distributed from all upland cotton chromosomes (Fig. 2c). Additionally, the highest densities of LncRNAs and mRNAs were mapped to the At-sub genome A05 and A11, whereas the least densities were detected in At-sub genome A02 and A04. In the Dt-sub genome, the highest densities of LncRNAs were detected in chromosome D11, and mRNAs were found in chromosomes D05 and D11. Upland cotton LncRNAs had fewer exons than that in mRNAs (protein-coding genes) (Fig. 2d) and LncRNAs and mRNAs had unequal distribution across the upland cotton chromosomes.

### Differentially expressed LncRNAs at different stages of cotton fiber development

**Fig. 3:**
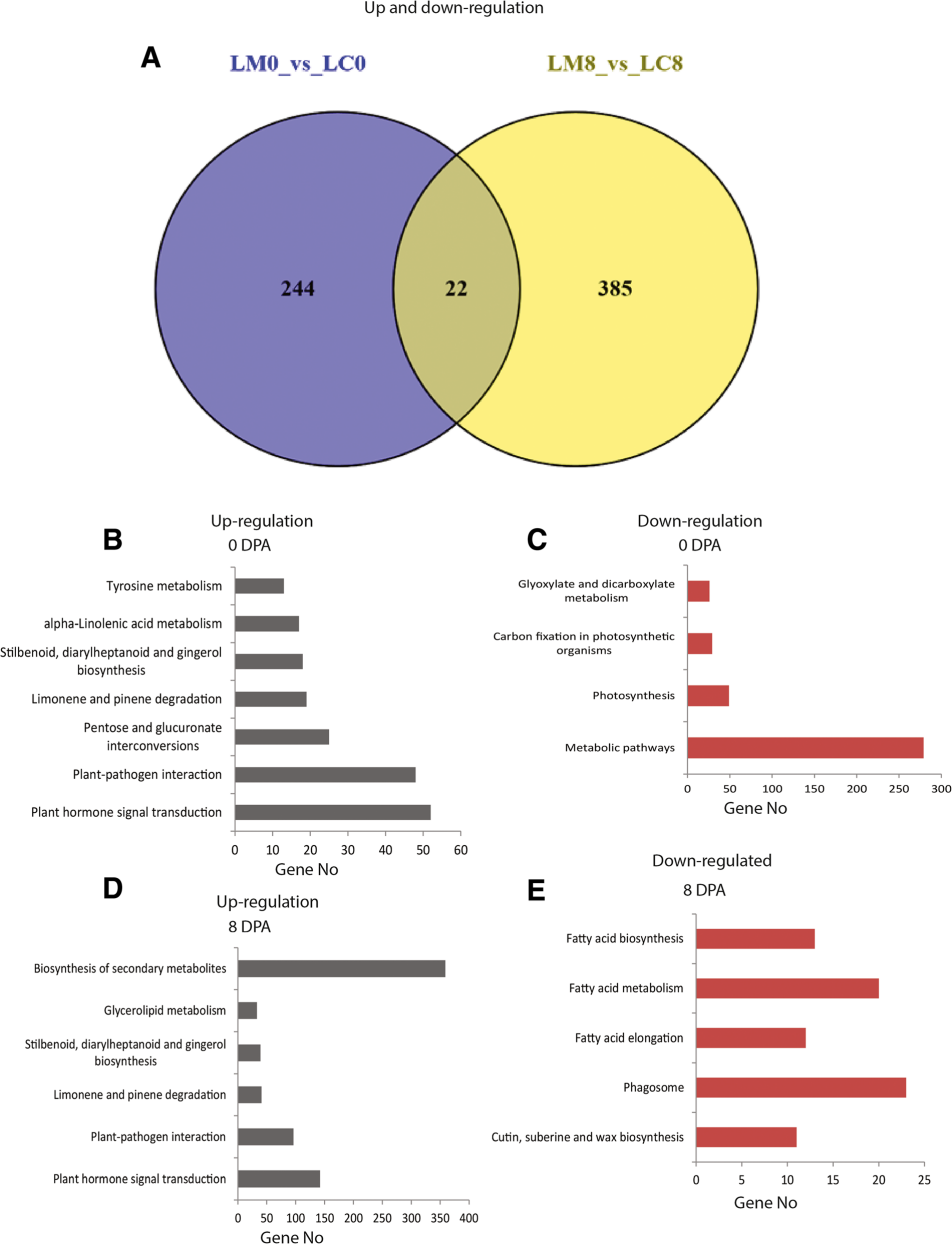
Differentially expressed LncRNAs expressed in Ligon-lintless-1 and wild-type. **a** A Venn diagram showing LncRNAs that was up and down-regulated in Ligon-lintless-1 and wild-type. **b** and (**c**) KEGG enrichment analysis of differentially expressed LncRNA-targets genes was up-regulated at 0 and 8 DPA, respectively. **d** and (**e**) KEGG enrichment analysis of differentially expressed LncRNA-target genes were analyses of differentially expressed LncRNA-target genes were down-regulated at 0 and 8 DPA, respectively

We examined the expression levels of LncRNAs related to cotton fiber development at 0 and 8 DPA using FPKM. LncRNAs were expressed at different levels in fiber development. To address whether the expression pattern of the LncRNAs is linked to a specific stage of cotton fiber development, we analyzed the differential expression levels of LncRNAs of each transcript between Ligon-lintless-1 mutant and near isogonic wild-type. At 8 DPA, higher numbers of LncRNAs were differentially expressed in Ligon-lintless-1 mutant than at 0 DPA (Additional file 2: Table S2 and Additional file 3: Table S3). We also identified differences in the expression profiles of LncRNAs and other transcript groups in the Ligon-lintless-1 and wild-type samples (Fig. 1b). The LncRNAs expression profiles in cotton fiber development were carried out by Cuffdiff which provided statistical procedures of determining the differential expression in a digital transcript of LncRNAs expression data using a model based on the negative binomial distribution [37]. A total of 266 and 407 LncRNAs were differentially expressed at 0 and 8 DPA respectively (Fig. 3a). Among them, 222 and 241 LncRNAs were up-regulated and 44 and 166 LncRNAs were down-regulated at 0 and 8 DPA respectively, which gave an indication of LncRNAs being either positively or negatively involved in the initiation and elongation stages of fiber development. In addition, there were more LncRNAs up-regulated than down-regulated in Ligon-lintless-1 mutant, which further giving proof of their negative role in fiber development. Only 22 common LncRNAs were found at 0 and 8 DPA while the rest, 244 and 385 LncRNAs were stage-specific to 0 and 8 DPA, respectively (Fig. 3a). More recently, actin-1 gene, GhACT_LI1 (Gh_D04G0865), has been found to be regulating fiber development [42], in references to this gene we found one LncRNA, LNC_012557 with differential expression only at 0 DPA. LNC_012557 had a higher transcription level in Ligon-lintless-1 but not in wild-type.

### Common differentially expressed LncRNAs in cotton fiber development

Cotton fiber initiation and elongation are key stages to determine the number and length of the fiber cell development. Lint of cotton fiber is supposed to appear on the day of anthesis (0 DPA) and continuously elongate until three weeks after the day post anthesis [9]. To predict putative functional LncRNAs related to cotton fiber development during initiation and elongation stages, the expression of 22 common LncRNAs were found at 0 and 8 DPA (Fig. 3a). The result showed that 19 LncRNAs were up-regulated in Ligon-lintless-1 mutant as compared to the wild-type during initiation stage of cotton fiber (0 DPA) while only 3 LncRNAs (LNC_017608, LNC_012210 and LNC_007516) were down-regulated in cotton fiber development at 0 DPA (Table 1). Specifically, these LncRNAs might be needed to maintain normal fiber development in wild-type. However, 18 LncRNAs were down-regulated at 8 DPA and only 4 LncRNAs (LNC_007516, LNC_008336, LNC_017132 and LNC_017608) were up-regulated during the elongation stage of fiber development at 8 DPA. The expression of these LncRNAs might in part trigger the early cessation of cotton fiber development in the Ligon-lintless-1 mutant. These LncRNAs have significantly higher transcription levels in normal cotton fiber than in mutant cotton fiber at 8 DPA (q-value < 0.05), but lower in transcription levels in Ligon-lintless-1 mutant than wild-type at 0 DPA (q-value < 0.05) (Table 1).

**Table 1:** Common differentially expressed LncRNAs in Ligon-lintless-1 mutant as compared to wild-type during cotton fiber development

LncRNAs ID	log2 (fold change 0 DPA Li1/WT)	Q value	log2 (fold change 8 DPA Li1/WT)	Q value
LNC_002075	2.41	5.00E-05	−1.87	0.00045
LNC_015258	2.49	0.0001	−1.76	0.01705
LNC_007516	−1.59	0.00015	5.54	0.0026
LNC_015379	2.75	0.00015	−2.33	0.00035
LNC_015166	2.46	5.00E-05	−1.57	5.00E-05
LNC_012210	−1.88	0.00355	−1.89	0.0186
LNC_015540	3.20	0.0059	−1.96	5.00E-05
LNC_014831	1.52	5.00E-05	−2.12	5.00E-05
LNC_010975	2.93	5.00E-05	−2.17	0.00085
LNC_001352	3.49	5.00E-05	−3.29	5.00E-05
LNC_008874	2.17	0.00435	−1.62	0.00395
LNC_001734	3.81	5.00E-05	−3.08	0.00035
LNC_001543	1.56	0.0086	−1.90	0.0042
LNC_008391	2.97	5.00E-05	−2.52	5.00E-05
LNC_008336	2.51	0.00105	3.27	0.00485
LNC_008289	1.75	0.00025	−1.88	0.0005
LNC_008291	1.76	5.00E-05	−1.85	5.00E-05
LNC_006271	1.98	0.00025	−1.61	0.00045
LNC_017132	1.60	0.0154	inf	0.00305
LNC_017608	−1.84	0.0001	2.56	5.00E-05
LNC_005974	1.53	0.00385	−2.52	0.0001
LNC_003053	1.84	5.00E-05	−1.84	5.00E-05

### Functional analysis of differentially expressed LncRNAs

**Fig. 4:**
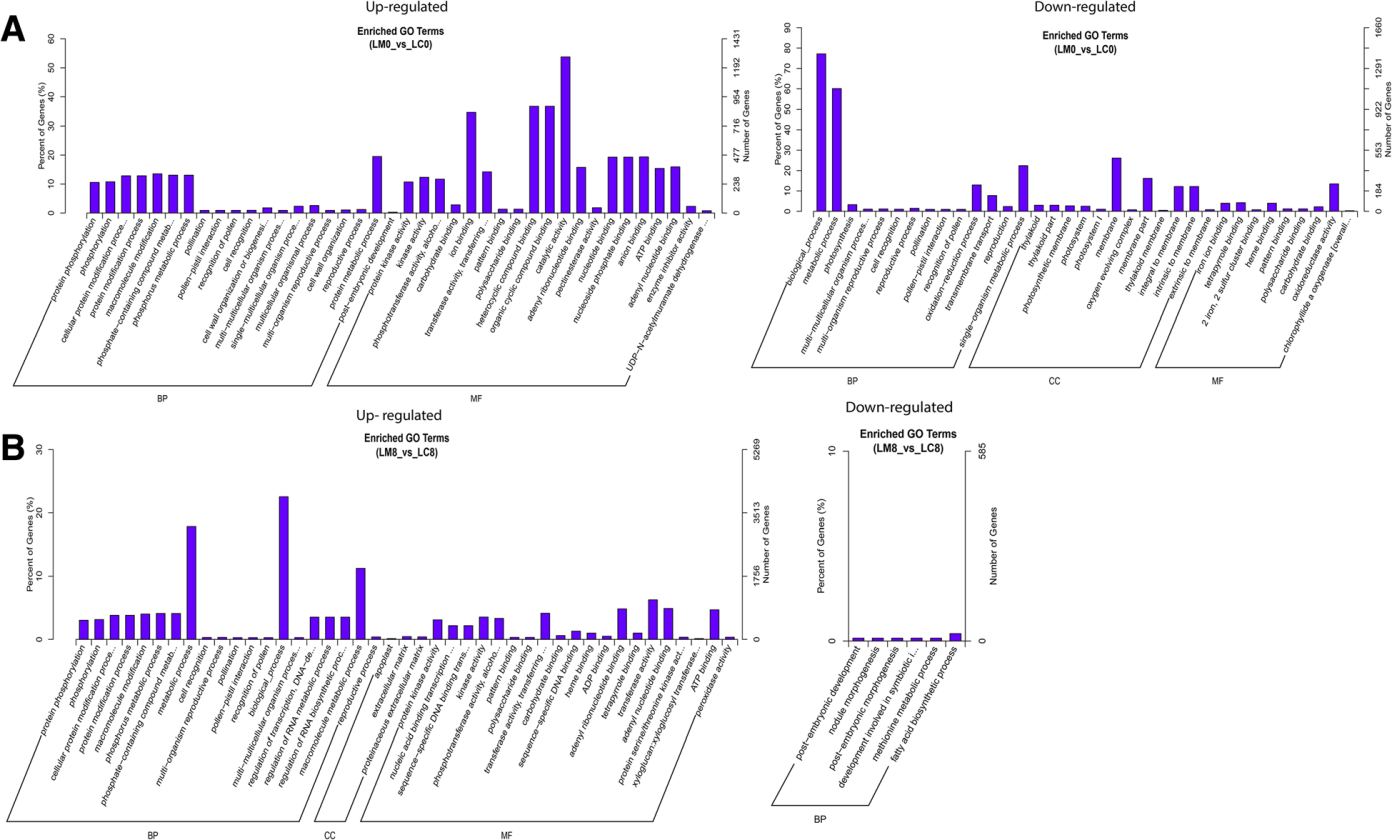
Gene ontology (GO) enrichment analysis of LncRNA-target genes. **a** The results of GO analysis based on differentially expressed LncRNAs targets genes were up and down-regulated at 0 DPA. **b** The results of GO analysis based on differentially expressed LncRNAs targets genes were up and down-regulated at 8 DPA

To investigate the specific functions of LncRNAs, we systematically predicted the possible targets of LncRNAs based on co-location and co-expression. According to the genome position of the LncRNAs and mRNAs (co-located), the nearest mRNAs around each LncRNA at downstream and upstream locations within 10 kb were investigated. The co-expressed genes were selected based on their expression correlation coefficients for the gene ontology (GO) enrichment analyses. The targets of differentially expressed LncRNAs were classified into different groups by gene ontology annotations as biological process, molecular function and cellular component. The GO analysis of LncRNAs targets based on co-location and co-expression at 0 and 8 DPA showed that some significantly enriched genes were mainly involved in the biological processes, such as protein phosphorylation, phosphorylation, cellular protein modification, protein modification and macromolecule modification, which were up-regulated in Ligon-lintless-1 mutant as compared to wild-type. As can be seen, the biological process and metabolic process were up-regulated at 8 DPA and down-regulated at 0 DPA (Fig. 4a & b). Phosphorylation is a process mediated by protein kinases to activate critical cellular pathways such as metabolism, cell division and cell differentiation during initiation stage in cotton fiber development [43]. Interestingly, Fatty acid biosynthetic process, methionine metabolic process, development involved in symbiotic interaction, post-embryonic morphogenesis, nodule morphogenesis and post-embryonic development were all down-regulated at 8 DPA in the Ligon-lintless-1 mutant (Fig. 4b). This result indicated clearly that these genes involved in biological process and metabolic process during the elongation stage might be responsible for the early cessation of fiber development. In the molecular function categories, kinase activity, protein kinase activity, phosphotransferase activity and transferase activity were significantly up-regulated in cell fiber development at 0 and 8 DPA. Protein kinase activity plays an important role in signal transduction through phosphorylation process during cotton fiber development [44]. Catalytic activity, heterocyclic compound binding, organic cyclic compound binding, carbohydrate binding, polysaccharide binding and pattern binding were highly up-regulated in Ligon-lintless-1 at 0 DPA (Fig. 4a). For the molecular component, membrane, membrane part, integral to membrane, thylakoid intrinsic to membrane and thylakoid part were down-regulated at the initiation stage (0 DPA), and only three gene ontology of apoplast, extracellular matrix and proteinaceous extracellular were up-regulated at 8 DPA (Fig. 4a & b).

We further applied all differentially expressed LncRNAs for the possible target genes using KEGG. The pathway analysis of co-located and co-expressed genes was enriched in plant hormone signal transduction, plant-pathogen interaction, pentose and glucuronate interconversions, limonene and pinene degradation, stilbenoid, diarylheptanoid and gingerol biosynthesis, alpha-linolenic acid metabolism and tyrosine metabolism pathways, which were up-regulated in the Ligon-lintless-1 mutant at 0 DPA. On the other hand, metabolic pathways, photosynthesis, carbon fixation in photosynthetic organisms, glyoxylate and dicarboxylate metabolism were down-regulated in Ligon-lintless-1 mutant at 0 DPA (Fig. 3b & c). At 8 DPA, the co-located and co-expressed genes were mainly involved in biosynthesis of secondary metabolites, plant hormone signals transduction, plant-pathogen interaction, limonene and pinene degradation, stilbenoid, diarylheptanoid and glycerolipid metabolism were up-regulated in Ligon-lintless-1. In contrast, the phagosome (23 genes), fatty acid metabolism (12 genes), fatty acid elongation (21 genes), fatty acid biosynthesis (13 genes) and cutin, suberine and wax biosynthesis (11 genes) were down-regulated at 8 DPA in the mutant cotton (Fig. 3d & e). These results suggested that the regulation of hormones and some enzymes catalyze metabolic processes, such as fatty acids, biosynthesis of secondary metabolites, transport process and glycerolipid metabolism. They may possibly play an important role during initiation and elongation stages of cotton fiber development. Based on the GO and KEGG analysis, we identified some potentially key target genes which were enriched in fatty acid biosynthesis, fatty acid metabolism, fatty acid elongation and transport process were down-regulated in Ligon-lintless-1 as compared to the wild-type during the elongation stage of cotton fiber development. These possible target genes provide new insight into the role of LncRNAs in cotton fiber developmental stages. In addition, plant hormone metabolism could also have effect on cotton fiber development at elongation stage, for example, a large number of LncRNAs were found to be involved in the metabolism of auxin, ethylene, abscisic acid (ABA) and gibberellin [45]. The majority of LncRNAs involved in the above hormones metabolism were found to be up-regulated in Ligon-lintless-1 mutant.

### Interaction determination between miRNAs and LncRNAs

**Fig. 5:**
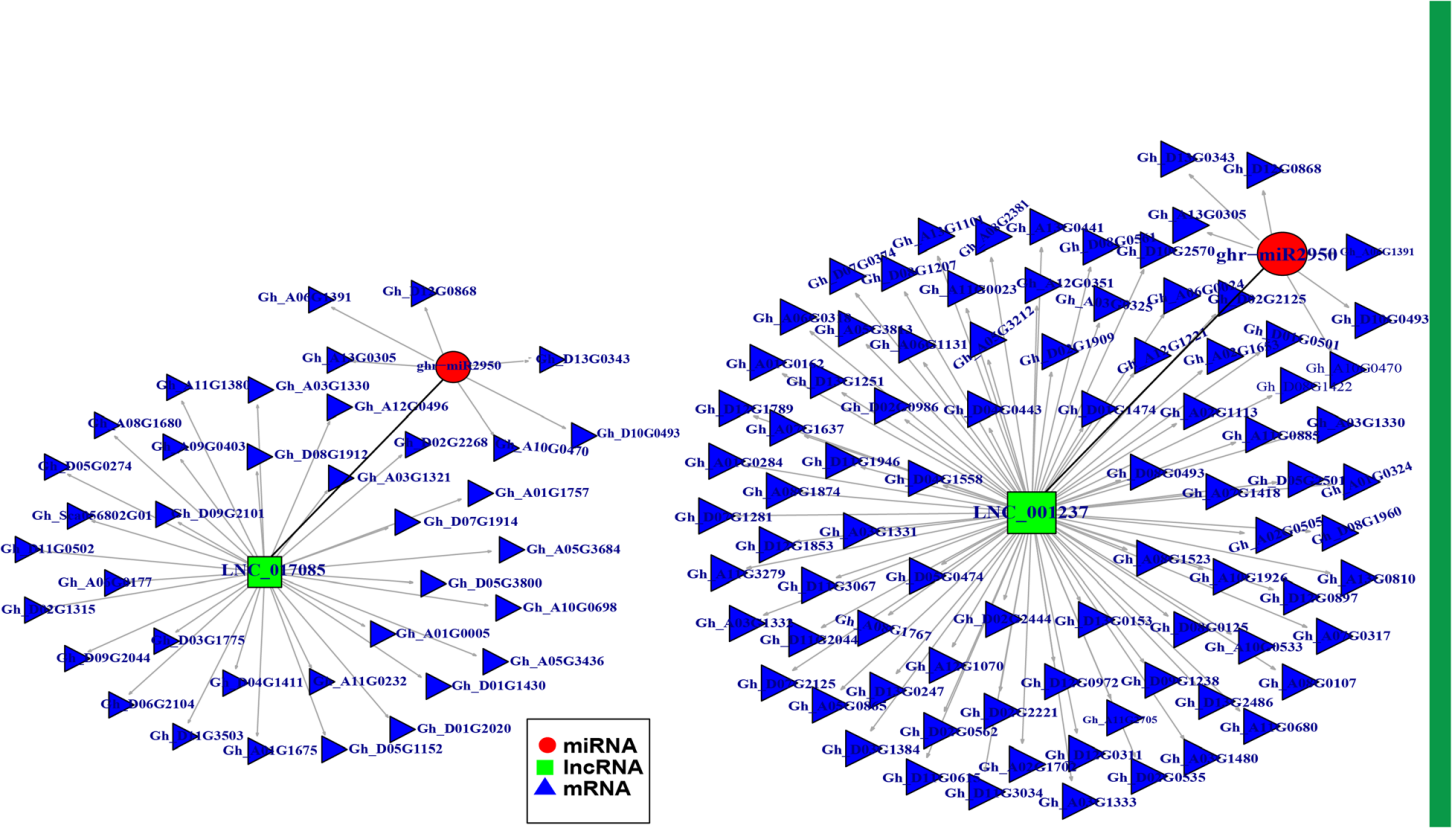
Interaction network among LncRNA, miRNA and mRNAs. The interaction network includes 2 LncRNAs (LNC_001237 and LNC_017085), mRNA and miRNAs (ghr-miR2950). Green square nodes represented LncRNAs, red circle nodes represented miRNAs and blue triangle nodes represented mRNAs

The interaction between miRNAs and LncRNAs is important for the determination of functional patterns of LncRNAs because of the destabilization and repressive nature of miRNAs [46]. LncRNA functions and stabilities can be disrupted by miRNAs [47]. To examine LncRNAs and miRNAs interactions, the differentially expressed LncRNAs were submitted to the psRNATarget website in order to obtain the miRNAs specifically targeting the various differentially expressed LncRNAs [48]. Out of 673 LncRNAs, 233 were found to be targeted by 58 miRNAs (Additional file 4: Table S4). A number of miRNAs detected such as miR156, miR160, miR162, miR164, miR166, miR167, miR169, miR172, miR390, miR393, miR394 and miR156 were found to have temporal differential expression patterns with the peak at − 2 DPA [49]. In cotton, it was previously observed that miR166, miR167, miR172 and miR2949 were highly expressed in the ovule [50]. Twenty (20) LncRNAs were found to be either destabilized or repressed by miR156; this could possibly explain their role in fiber development in mutant cotton. This result is consistent with the expression analysis of miR156 and others in fiber initiation at − 2 DPA in Xu-142 (mutant) and Xu-142 (wild type), in which miR156, miR157, miR159 and miR160 among others were found to be significantly up-regulated in mutant cotton as compared to the wild-type [51]. Similarly, miR156 was found to have a temporal differential expressions pattern with the peak at − 2 DPA. In this study, we identified 14 miRNAs which were differentially expressed during cotton fiber development in Ligon-lintless-1 as compared to wild-type. Among these miRNAs, ghr-miR2950 (targeting to fatty acid hydrolase), ghr-miR7506, ghr-miR7504a (targeting to cleave carbohydrate-Active enzymes family), ghr-miR7484a (targeted to cleave Ring/U-box domain containing protein) and ghr-miR7502, ghr-miR7499, ghr-miR7492a, ghr-miR399e (targeted to cleave AP2/B3-like transcriptional factor), ghr-miR156c (targeted to cleave squamosal promoter-binding protein), ghr-miR169b and ghr-miR7496a were down-regulated in Ligon-lintless-1 whereas ghr-miR7510a (targeted to cleave peroxidase super family protein) and ghr-miR7506, ghr-miR7498 and ghr-miR7497 were up-regulated in Ligon-lintless-1 during cotton fiber development, indicating that cotton mutation strongly disturbed their expressions. In contrast, ghr-miR7510b (targeted to cleave peroxidase super family protein) and ghr-miR7492a were up-regulated during the initiation stage (0 DPA), and down-regulated during elongation stage (8 DPA) in the Ligon-lintless-1 mutant (Additional file 5: Table S5). In addition, ghr-miR2950 was predicted to target 8 down-regulated LncRNAs. In this context, the two, LNC_017085 and LNC_001237 were significantly down-regulated in Lingon-lintless-1 mutant by more than 5-fold and 4-fold, respectively (Additional file 5: Table S5). The interaction between the two aforementioned LncRNAs and ghr-miR2950 is predicted to be involved in the destabilization of fatty acid hydroxylase (Fig. 5).

### Interaction between LncRNAs and mRNAs

To further investigate the differential expression of LNC_017085 and LNC_001237 with high potential targets of many mRNAs, we only focused on the significantly expressed mRNA with a high potential role in fiber development (Fig. 5). These two LncRNAs were found to target several mRNAs related to metabolic processes of lipid-transfer protein, tubulin, beta-6 tubulin, tubulin alpha-3, tonoplast intrinsic protein, sucrose nonfermenting 3, SAUR-like auxin-responsive protein family, cytochrome P450, actin depolymerizing factor, Glycosyl hydrolase, FASCICLIN-like, pectin methylesterase inhibitor and galacturonosyltransferase, which were down-regulated in Li- 1 mutant at 8 DPA. Recently, the growing evidence suggests that cytoskeleton is involved in regulating cotton fiber development [42]. It has been revealed that fiber elongation is coupled with dynamic changes in the structure of the actin cytoskeleton and microtubules [42]. Actin depolymerizing factors were significantly down-regulated by more than 2-fold in Ligon-lintless-1 mutant. The low expression pattern of Actin depolymerizing factor gene affects cotton fiber development [52]. The expression of tubulin, beta-6 tubulin and tubulin alpha-3 were consistent with earlier reports [17, 53] in which both reports showed that tubulin, beta-6 tubulin and tubulin alpha-3 were down-regulated by more than 3-fold in the Li-1 mutant at elongation stage of fiber. Tubulin is a vital cytoskeleton protein with significance in cotton fiber elongation [54]. Several genes belonging to cell wall metabolism such as Glycosyl hydrolase, *FASCICLIN*-like, pectin methylesterase inhibitor, lipid-transfer protein and galacturonosyltransferase were down-regulated in Ligon-lintless-1 mutant during fiber elongation stage.

### Expression verification of candidate LncRNAs involved in cotton fiber development

**Fig. 6:**
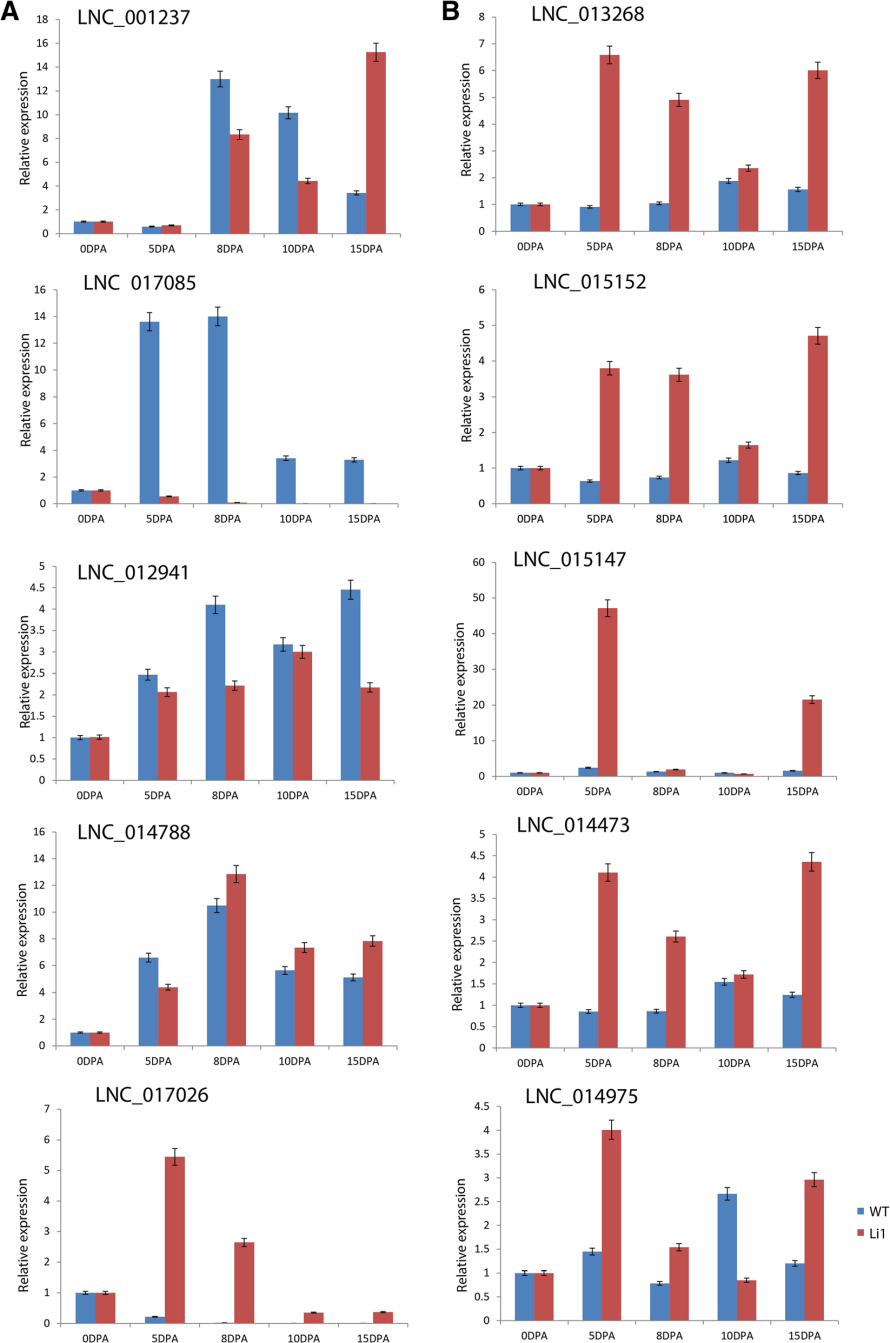
RT-qPCR validation of RNA-seq data on the accumulation of 10 LncRNA related to fiber development. Down-regulated LncRNAs (**a**) and up-regulated LncRNAs (**b**) were highly expressed in Ligon-lintless-1 mutant and wild-type during cotton fiber development based on RNA-seq data.

Long non-coding RNAs play a significant role in many plants in terms of growth, development and adaptation mechanisms, such as flower development [55], sexual reproduction [56], fruit ripening [57], fiber development [28], biotic stress [58] and abiotic stress responses [59]. To examine whether these differentially expressed LncRNAs had a role in fiber development during the elongation stage, 10 LncRNAs were selected based on their expression levels in RNA-seq. Five down-regulated LncRNAs 5 up-regulated LncRNAs at 8 DPA were selected and validated with RT-qPCR. The down-regulated LncRNAs were LNC_001237, LNC_017085, LNC_017026, LNC_012941 and LNC_014788.LNC_013268, LNC_015152, LNC_015147, LNC_014473 and LNC_014975 were up-regulated at 8 DPA (Fig. 6a and b). The results of the down-regulated LncRNAs validation, showed a deviation from the RNA-seq, in which three, LNC_001237, LNC_017085, and LNC_012941 were highly expressed in the wild-type at 5, 8 and 10 DPA but LNC_017026 and LNC_014788 had low expression in wild-type compared to the mutant type at 8, 10 and 15 DPA (Fig. 6a). The down-regulation of LNC_001237 and LNC_017085 could be attributed to the destabilizing or repressive nature of ghr-miR2950. The up-regulated LncRNAs such as LNC_013268, LNC_015152, LNC_015147, LNC_014473 and LNC_014975 were highly expressed in Ligon-lintless-1 mutant but showed low expression levels in the wild-type at 5, 8 and 15 DPA. This result is consistent with the whole RNA-seq analysis in which all the 5 LncRNAs (LNC_001237, LNC_017026, LNC_017085, LNC_012941 and LNC_014788) were significantly expressed in Ligon-lintless-1 compared to the wild-type at 8 DPA. Accordingly, the up-regulated LncRNAs in Ligon-lintless-1 mutant might be responsible for the early cessation of the fiber elongation stage (Fig. 6b). RT-qPCR analysis showed that all of the up-regulated LncRNAs were highly expressed in Ligon-lintless-1 mutant but not in wild-type, this validates the negative role of LncRNAs in cotton fiber development during elongation stage (Fig. 6b).

## Discussion

Cotton fiber initiation and rapid elongation stages are crucial stages with greater impact on fiber quantity and fiber length. It is essential to understand the various molecular pathways involved in the development of cotton fiber. Several proteins or genes have been identified with functional roles in fiber development [16, 20, 28, 46, 47]. However, the molecular elements which are responsible for early cessation of the fiber elongation process in Ligon-lintless-1 mutant are still largely not understood. LncRNAs were first discovered in 1991, highlighting certain regulatory pathways involved in different functions of the organism [48]. In recent years, there have been an upsurge in the number of studies on LncRNAs in relation to their roles in various biological processes across plants and animals [21, 24, 25, 27, 28, 49–52, 54, 60–63]. This study aimed at carrying out systematic identification and analysis of differentially expressed LncRNAs in Ligon-lintless-1 mutant and wild-type of *G. hirsutum*. In general, the expression levels of LncRNAs showed significantly lower than that in the protein-coding transcripts (Fig. 1b & c), which conformed with a previous report [28]. Transcriptome analysis in *Gossypium arboreum* and *Gossypium barbadense* showed that the two types of LncRNAs, the LincRNAs and antisense (LncRNAs) had less exons [28, 64]. The total number of long non-coding RNAs identified were fewer compared to earlier findings in *G. barbadense* [28], the difference in the number of LncRNAs, could possibly be due to the genome differences. To further understand the roles of LncRNAs in Ligon-lintless-1 mutant and wild-type, expression profiling was done at 0 and 8 DPA. Additionally, the results of this study revealed that the majority of differentially expressed LncRNAs were precisely expressed at the specific stage of development, indicating the functional divergence of LncRNAs in cotton fiber development. The expressions of LncRNAs are temporal-specific, highly conserved LncRNAs and are actively regulated and do function predominantly in embryonic development reported on the study of the evolution of LncRNAs repertoires and expression patterns in tetrapods [65]. Interestingly, we found that the LncRNAs were time specific in terms of their expression patterns. In rice, the LncRNAs have unique characteristics compared to those of Arabidopsis in terms of their expression patterns. These groups of LncRNAs have tissue and or stage specificity in their expression profiling patterns [56]. Expression levels of a number of LncRNAs in Ligon-lintless-1 mutant were significantly varied from those in wild-type at 0 and 8 DPA (Additional file 3: Table S3).

Gene Ontology and KEGG pathway analyses based on differentially expressed LncRNAs on target genes identified fatty acid biosynthesis as an inhibitory process related to cotton fiber development (Figs. 3e and 4b). Fatty acid biosynthesis and fatty acid elongation were down-regulated in the Ligon-lintless-1 mutant at 8 DPA contrary to the previous finding in which fatty acid biosynthesis and fatty acid elongation were found to be up-regulated during cotton fiber elongation [66, 57]. Fatty acid biosynthesis and fatty acid elongation do promote trichome cell elongation through stimulation of ethylene biosynthesis [67]. The enzymes that catalyze fatty acid metabolism in Ligon-lintless-1 mutant might indirectly or directly be involved in cotton fiber elongation. This finding is in agreement with a previous report which stated that the majority of genes involved in the synthesis of very long chain fatty acid biosynthesis were down-regulated in the Ligon-lintless-1 mutant [59]. Gene Ontology and KEGG pathway analyses based on differentially expressed LncRNAs on target genes identified fatty acid biosynthesis as an inhibitory process related to cotton fiber development (Figs. 3e and 4b).

In addition, a greater percentage of the differentially expressed LncRNAs in Ligon-lintless-1 were specifically targeted by miRNA, which is an indication of their possible roles in the development process of cotton fiber. Privous several studies reported the influence of miRNAs in cotton fiber development [31, 50]. The high expression level of ghr-miR2950 in the wild-type may play a key role in the elongation stage of fiber. The expression analysis of RNA seq showed that ghr-miR2950 was significantly expressed in wild-type as compared to Ligon-lintless-1 at 8 DPA [31]. It was found that ghr-miR2950 exhibited a higher expression level during the fiber elongation stage than at ovulation stage [49]. In plant cell division, ghr-miR2950 was found to be highly expressed in tall-culm mutant cotton, possibly explaining the plant height [60]. The finding of this research that fatty acid hydroxylase was the putative target of miR2950. Fatty acid hydroxylase was significantly up-regulated at 10 DPA compared to the wild type, further explaining its significant contribution to fiber length in domesticated cotton, [61]. Ring/U-box domain containing protein had an interaction with ghr-miR7484a, which was down-regulated in Ligon-lintless-1. Previously, it was indicated that, ghr-miR7484 had a role in regulating cotton fiber development through targeting MYB in cotton [50]. This implied that ghr-miR7484a might be involved in regulation of cotton fiber development during elongation stage. RING/U-box super family proteins were involved in the transitional developmental stages of cotton fiber [62]. The expression pattern of ghr-miR7510b was found to target peroxidase super family protein which was up-regulated at 0 DPA but down-regulated at 8 DPA in Ligon-lintless-1 mutant. Peroxidase was found to directly correlates to the accumulation of reactive oxygen species which related to cotton fiber elongation [63]. The high expression levels of ghr-miR7510b and ghr-miR7492a in Ligon-lintless-1 at 0 DPA were to trigger the initiation of cotton fiber while their lower expression levels during elongation stages at 8 DPA initiated the cessation of cotton fiber elongation.

To further investigate the differential expression of LNC_017085 and LNC_001237 with high potential targets of many mRNAs, we only focused on the significantly expressed mRNA with a high potential role in fiber development. This result provides a strong indication that genes related to cell wall metabolism could be playing key roles in cotton fiber development. Pectin methylesterase is the enzyme which is involved in cell wall structure of plants through deposition of the pectin [68]. In cotton, pectin methylesterase could play an important role in cell fiber wall structure of cotton fiber through pectin deposition [69]. Lipids are involved in the regulation of cotton fiber elongation stage through facilitating the transportation of phosphatidylinositol [70]. During the elongation stage of cotton fiber development, the continuous synthesis of lipids and protein transport are the key steps to promote the dynamic change in enlargement of plasma membrane and vacuoles [71]. Both RNA-seq analysis and RT-qPCR result in Ligon-lintless-1 mutant and wild-type provide strong evidence of LNC_001237, LNC_017085 and ghr-miR2950 being integral molecular elements that are likely involved in the various pathways of cotton fiber development.

## Conclusion

The findings of this study provide novel insights into the better understanding of LncRNAs regulatory role in the molecular pathways regulating cotton fiber development. Further research on designing and transforming the LncRNAs will help not only in understanding their functions but will also help in improving fiber quality.

## Methods

### Plant material and RNA extraction

*G. hirsutum*, Ligon-lintless-1 mutant (Li2Li2) and its wild-type (li2li2) were derived from self-pollinated Ligon-lintless-1plants of at least more 6 generations in our lab [72]. The Ligon-lintless-1 seeds were provided by Kohel (USA) and used in this research study for LncRNA analysis. The two cotton genotypes were grown in the experimental field at the Cotton Research Institute, Chinese Academy of Agricultural Sciences (ICR, CAAS) Anyang city, Henan province, China. One day before anthesis, cotton flowers were tied and tagged for self-pollination. A total of 198 Ligon-lintless-1 mutant Li2Li2 plants and 128 wild-type li2li2 plants were used for samples collection. Cotton bolls were collected at 0 (ovules) and 8 DPA (Fiber) during cotton fiber development. Biological triplicates for each sample were collected during cotton fiber development at 0 and 8 DPA from Ligon-lintless-1 and wild-type. Briefly, cotton fibers separated from the developing ovules using glass bead shearing method/liquid nitrogen. The harvested samples were rapidly frozen in liquid nitrogen and kept at − 80 °C until analysis. Cotton fibers were initial ground in liquid nitrogen using a mortar and pistil, and the gridded samples were rapidly transferred into tubes (2 ml micro) with 700 μL lysis/binding buffer. RNA samples were extracted using TRIzol (Invitrogen). RNA contamination and degradation were evaluated on 1% agarose gel. RNA purity and integrity were tested using the NanoDropPhotometer® spectrophotometer RNA and Nano 6000 Assay Kit of the Bioanalyzer 2100 system, separately. Finally, the concentration of RNA was checked using Qubit® RNA Assay Kit in Qubit® 2.0 Flurometer.

### Library preparation for lncRNA sequencing

A total of 3 μg RNA per sample was used as input material for the RNA sample preparations. At the initial stage, ribosomal RNA was removed by Epicenter Ribo-zero™ rRNA Removal Kit (Epicenter, USA), and rRNA free residue was cleaned up by ethanol precipitation. The sequencing libraries were prepared using the rRNA-depleted RNA by NEBNext® Ultra™ Directional RNA Library Prep Kit for Illumina® (NEB, USA) following the manufacturer’s specifications. Fragmentation was performed using divalent cations under raised temperature in NEBNext First-Strand Synthesis Reaction Buffer. The first-strand cDNA was produced using random hexamer primer and M-MuLV Reverse Transcriptase (RNaseH-)and the second-strand cDNA production was subsequently conducted using RNase H and DNA polymerase I. In the reaction buffer, dTTP and dNTPs were changed by dUTP. The rest of the overhangs were successfully transformed into blunt ends through exonuclease/polymerase enzyme activities. After adenylation of 3’ ends of DNA fragments, NEBNext Adaptor with the hairpin loop organizations were ligated to make for hybridization. In order to identify cDNA fragments of specially 150~200 base pairs, the library fragments were cleaned using AMPure XP system (Beckman Coulter, Beverly, USA). Then 3 μl USER Enzyme (NEB, USA) was used with size selected adaptor-ligated cDNA at 37 °C for 15 min followed by 5 min at 95 °C before PCR. PCR was then performed with Phusion High-Fidelity DNA polymerase using universal PCR primers and index (X) Primer. The final, harvests were purified (AMPure XP system) and library quality was assessed on the Agilent Bioanalyzer 2100 system.

### Bioinformatic identification of upland cotton LncRNAs

Raw data of fastq format were processed through in-house perl scripts to obtain clean data. The clean data (clean reads) were obtained by removing the adapter and low-quality reads (quality score > Q20). The clean datasets with high quality were mapped independently with reference to the upland cotton genome, using bothTophat2 (v2.0.9) software [35] and Bowtie 2 (version 2.2.9) [36]. Reference genome and gene model annotation files were downloaded from genome website (http://mascotton.njau.edu.cn/html/Data) [34]. Cufflinks (v2.1.1) [37] and Scripture (beta2) [38] in a reference-based approach were applied to assemble the transcriptomes which were generated by Tophat2 (v2.0.9) and Bowtie 2. All transcriptomes from four cotton fiber development samples were pooled and merged to perform a comprehensive transcriptome using Cuffmerge. The CUFFCOMPARE procedure was used to compare all the transcript assemblies to the upland cotton genome. Then we adapted five steps of filtrations to identify LncRNAs from transcriptome assemblies: (1) transcripts assembled by two methods were detected in less than two samples were discarded; (2) transcripts without strand sense information and short transcripts without mapping coverage of more than half of the transcript length were removed; (3) transcripts overlapped with mRNA on the same strand, transcripts with FPKM (fragments per kilo-base of exon per million fragments mapped) ≥ 0.5 (2 for single-exon transcripts) and short transcripts with length less than 200 bp were eliminated; (4) transcripts with potential encoding hit were excluded by CPC (Coding Potential Calculator) and CNCI (Coding-Non-Coding Index) [40] and (5) the remaining transcripts were queried against the Pfam scan (Pfam databases) to remove all transcripts with known protein-coding domains (E-value 0.001) [41]; transcripts without capability of coding proteins were considered as LncRNAs with length ≥ 200 bp.

### Gene expression analysis

Cuffdiff (v2.1.1) was used to calculate FPKMs of both LncRNAs and coding genes in each sample [37]. Gene FPKMs were calculated by summing the FPKMs of transcripts in each gene group. FPKM value is calculated based on the length of the fragments and reads count mapped to this fragment.

### Target gene prediction

Determination of target gene prediction of LncRNA was acting on neighboring genes (co-location). We searched the neighboring coding genes around every LncRNA at upstream and downstream locations within 10 kb to investigate their possible functions. Co-expression of the target gene prediction in LncRNA was identified by their expression level. The prediction method of co-location and co-expression of the target genes were done according to the previous studies [73, 74]. Pearson correlation coefficient (r_p_) was applied to elucidate the expression relationship between LncRNAs and mRNAs pairs using R tool. LncRNAs co-expressed target mRNAs (genes) were identified with (R^2^) ≥ 0.95. We clustered the genes from various samples with WGCNA [75] for the search of common expression modules and the analysis of their function depending on the functional enrichment analysis.

### Differential expression analysis

Cuffdiff provides statistical routines for determining differential expression in a digital transcript or gene expression data using a model based on the negative binomial distribution [37]. Only transcripts that had FPKM more than 1 were considered to be expressed. The differentially expressed transcripts were identified with q-value < 0.05 and a fold-change ≥1.5 or ≤ − 1.5 between the Ligon-lintless-1 mutant and wild-type.

### GO and KEGG enrichment analysis

GO (Gene Ontology) analyses of the differential expression (DE) of genes or the LncRNA target genes were performed by the GO seq R package, where the length bias of each gene was corrected. GO term analysis was considered significant at a corrected *P*-value < 0.05 were. KEGG (Kyoto Encyclopedia of Genes and Genomes) pathway is a database resource to understand the functions of the biological organization. KOBAS software was used to test the statistical enrichment of the DE genes or the LncRNA target genes in KEGG pathways with corrected *P*-value < 0.05.

### Small RNA sequencing and processing

Small RNA libraries preparation and sequencing were conducted by LC Science (Houston, TX). Biological triplicates of RNA samples extracted from cotton fiber development at 0 and 8 DPA were combined together for preparation of Ligon-lintless-1 and wild-type microRNA libraries, separately. The micro RNA libraries were constructed using 1 μg of total RNA based on the TruSeq® microRNA sample preparation guide (Illumina). The general process comprised the following: firstly, the total RNA was ligated to RNA 3′ and RNA 5′ adapters. Secondly, the cDNA constructs were created by reverse transcription followed by PCR; the construction was based on the microRNAs ligated with 3′ and 5′ adapters. Thirdly, small cDNA fractions of length ranging between 22 nt and 30 nt were isolated by using 6% denaturing polyacrylamide gel electrophoresis. Finally, the cDNA construct was purified, and the library was validated. The libraries sequencing was done using the Illumina Hiseq 2500 platform.

### Identification of conserved and novel microRNAs

Clean reads were run through miRPlant software for identification of plant microRNAs from microRNA sequencing data [58]. *Gossypium hirsutum* TM-1 genome [34] was used for mapping reads with software’s default parameters. The miRPlant, predicted miRNAs were queried against the miRBase database (version 21, http://www.mirbase.org/) to identify conserved and reported earlier microRNAs. Matched sequences with no more than two mismatches were considered as candidate conserved or formerly reported miRNAs and were assigned to the corresponding miRBase family. Predicted microRNAs with more than 2 mismatches were considered as potential novel miRNAs; they lack sufficient similarity to assign to a microRNA family [76]. Statistical importance of differential expression levels of microRNAs in the RNA-seq data was calculated with the Audic and Claverie statistic using IDEG6 software [77].

### Interaction determination between miRNAs and lncRNAs

The interaction between miRNAs and LncRNAs and their potential roles in regulating gene expression were previously described in higher plants. Upland cotton LncRNAs were predicted as miRNA targets using the psRNATarget [78].

### RT-qPCR analysis

To examine the LncRNA expression patterns, cotton tissues from Ligon-lintles-1 and its wild-type were harvested at different stages of cotton fiber development such as 0, 5, 8, 10, and 15 DPA. High quality of RNA was treated by DNase I (TaKaRa, Japan) to eliminate contaminating DNA. The cDNA was generated from 2 μg of RNA in reaction volume of 20 μL using ReverTra Ace PCR-qRT kit based on the manufacturer’s requirement. RT-qPCR analysis was applied to test LncRNAs expression levels during developmental stages of cotton fiber. RT-qPCR experiment analysis was performed using the SYBER premix ExTaq kit (TaKaRa. Japan) and the Applied Biosystems 7500 Real-Time PCR system. SYBR Green was estimated the amplification targeted LncRNA. The housekeeping β-actin gene of the cotton was applied as a reference and primers were used for RT-qPCR. The following thermal cycler settings and LncRNA expression analysis were done according to salih et al. [79].

## Additional files

Additional file 1: **Table S1.** The numbers of annotated genes (mRNAs) and other transcript reads mapped to the TM-1 reference genome (XLSX 13 kb)

Additional file 2: **Table S2.** Differentially expressed LncRNAs in Ligon-lintless-1 and wild-type during cotton fiber development were expressed at 0 and 8 DPA. (XLSX 2671 kb)

Additional file 3: **Table S3.** Differentially expressed LncRNAs during cotton fiber development at 0 DPA in Ligon-lintless-1 and wild-type (XLSX 10464 kb)

Additional file 4: **Table S4.** LncRNAs were predicted as miRNA targets using the psRNATarget. (XLSX 27 kb)

Additional file 5: **Table S5.** Differentially expressed miRNAs in Ligon-lintless-1 mutant as compared to wild-type were expressed during cotton fiber development at 0 and 8 DPA. (XLSX 11 kb)

## Data Availability

All related data are available within the manuscript and its additional files.

## Abbreviations

bp: Bias pair
DPA: Day post anthesis
Li-1: Ligon-lintless-1
LncRNAs: Long non-coding RNAs
WT: Wild-type
